# Ex vivo gene editing and cell therapy for hereditary tyrosinemia type 1

**DOI:** 10.1097/HC9.0000000000000424

**Published:** 2024-04-26

**Authors:** Ilayda Ates, Callie Stuart, Tanner Rathbone, Mercedes Barzi, Gordon He, Angela M. Major, Vijay Shankar, Rachel A. Lyman, Sidney S. Angner, Trudy F.C. Mackay, Shanthi Srinivasan, Alton Brad Farris, Karl-Dimiter Bissig, Renee N. Cottle

**Affiliations:** 1 Department of Bioengineering, Clemson University, Clemson, South Carolina, USA; 2 Department of Pediatrics, Division of Medical Genetics, Alice and Y.T. Chen Center for Genetics and Genomics, Duke University School of Medicine, Durham, North Carolina, USA; 3 Department of Pathology, Texas Children’s Hospital, Houston, Texas, USA; 4 Department of Biochemistry and Genetics, Clemson University, Clemson, South Carolina, USA; 5 Center for Human Genetics, Clemson University, Greenwood, South Carolina, USA; 6 Department of Medicine, Digestive Diseases Division, Emory University School of Medicine, Atlanta, Georgia, USA; 7 Department of Pathology and Laboratory Medicine, Emory University School of Medicine, Atlanta, Georgia, USA; 8 Department of Medicine, Division of Gastroenterology, Duke University Medical Center, Durham, North Carolina, USA; 9 Department of Biomedical Engineering (BME) at the Duke University Pratt School of Engineering, Durham, North Carolina, USA; 10 Duke Cancer Center, Duke University Medical Center, Durham, North Carolina, USA; 11 Department of Pharmacology and Cancer Biology, Duke University Medical Center, Durham, North Carolina, USA

## Abstract

**Background:**

We previously demonstrated the successful use of in vivo CRISPR gene editing to delete 4-hydroxyphenylpyruvate dioxygenase (*HPD*) to rescue mice deficient in fumarylacetoacetate hydrolase (FAH), a disorder known as hereditary tyrosinemia type 1 (HT1). The aim of this study was to develop an ex vivo gene-editing protocol and apply it as a cell therapy for HT1.

**Methods:**

We isolated hepatocytes from wild-type (C57BL/6J) and *Fah*^−/−^ mice and then used an optimized electroporation protocol to deliver *Hpd*-targeting CRISPR-Cas9 ribonucleoproteins into hepatocytes. Next, hepatocytes were transiently incubated in cytokine recovery media formulated to block apoptosis, followed by splenic injection into recipient *Fah*^*−/−*^ mice.

**Results:**

We observed robust engraftment and expansion of transplanted gene-edited hepatocytes from wild-type donors in the livers of recipient mice when transient incubation with our cytokine recovery media was used after electroporation and negligible engraftment without the media (mean: 46.8% and 0.83%, respectively; *p*=0.0025). Thus, the cytokine recovery medium was critical to our electroporation protocol. When hepatocytes from *Fah*^*−*/*−*^ mice were used as donors for transplantation, we observed 35% and 28% engraftment for *Hpd*-Cas9 ribonucleoproteins and Cas9 mRNA, respectively. Tyrosine, phenylalanine, and biochemical markers of liver injury normalized in both *Hpd*-targeting Cas9 ribonucleoprotein and mRNA groups independent of induced inhibition of Hpd through nitisinone, indicating correction of disease indicators in *Fah*^*−*/*−*^ mice.

**Conclusions:**

The successful liver cell therapy for HT1 validates our protocol and, despite the known growth advantage of HT1, showcases ex vivo gene editing using electroporation in combination with liver cell therapy to cure a disease model. These advancements underscore the potential impacts of electroporation combined with transplantation as a cell therapy.

## Graphical abstract

**Figure:**
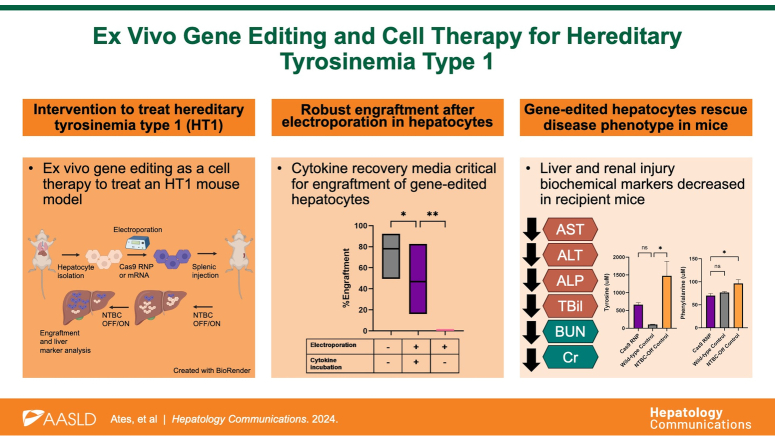

## INTRODUCTION

Hereditary tyrosinemia type 1 (HT1) is caused by a deficiency in fumarylacetoacetate hydrolase (FAH) resulting in acute liver failure, neurologic crisis, HCC, and early death.^1^ HT1 is treated with the drug 2-(2-nitro-4-trifluoro-methylbenzyol)-1,3 cyclohexanedione (NTBC), also known as nitisinone, to inhibit the enzyme 4-hydroxyphenylpyruvate dioxygenase (*Hpd*), and block the buildup of downstream toxic metabolites.^1^ NTBC is combined with dietary restrictions of tyrosine and phenylalanine to lower tyrosine levels and prevent disease symptoms. While this standard of care significantly decreases morbidity and mortality, patients with HT1 still face an elevated risk of HCC, especially in cases of late diagnosis or inadequate therapeutic compliance.^2^ Replacement of the diseased liver with a liver allograft from a healthy donor in OLT represents a curative final therapeutic resort, with the majority of transplants occurring in pediatric patients.^3^ However, the high risk of mortality, posttransplant complications, life-long immunosuppressive therapy, and organ shortages limit liver transplantation.^4–6^

CRISPR-Cas9–mediated gene editing to permanently disrupt therapeutic genes and reprogram metabolic pathways shows tremendous promise for treating many inherited metabolic diseases (IMDs) of the liver. However, delivering CRISPR components into target cells represents a grand challenge and is critical to achieving a therapeutic effect in patients. Adeno-associated viral vectors (AAVs) are the most commonly used delivery method for introducing CRISPR-Cas9 into animal models of human IMDs of the liver, such as hemophilia,^7^ alpha-1 antitrypsin deficiency,^8^ and HT1.^9^ Nonetheless, AAVs have immunogenicity risks due to pre-existing immunity against AAV capsids. AAVs activate cytotoxic CD8^+^ T-cell responses that cause loss of transduced hepatocytes and therapeutic failure.^10^ Anti-capsid neutralizing antibodies also contribute to pre-existing immunity and inhibit AAV transduction in animal models^11–14^ and humans.^15–18^ Modification of the AAV capsid has been proposed to overcome AAV immunity risks, but safety concerns still remain.^19,20^

Applying AAVs to deliver Cas9 is associated with additional limitations, such as potential integration into Cas9 on-target^21–23^ and off-target sites.^24^ Insertional mutagenesis by AAV vectors has been shown to cause HCC in experimental animal models.^25,26^ Since AAVs exist as stable episomes, there are concerns that persistent Cas9 expression increases off-target activity and genotoxicity.^27^ An additional barrier is the substantial prevalence of pre-existing Cas9 immunity in the human population, with up to 78% of individuals having anti-Cas9 IgG antibodies and Cas9-specific T cells.^28,29^ In a recent study by Li et al,^30^ AAVs containing CRISPR-Cas9 introduced into a host with pre-existing immunity led to cytotoxic T-cell elimination of gene-modified target hepatocytes in vivo, indicating that AAV delivery of CRISPRs is hampered by pre-existing Cas9 immunity.

These limitations can be avoided by ex vivo electroporation, a physical nonviral method that applies high-voltage currents to deliver biomolecules into a wide array of cell types at all cell cycle stages.^31,32^ In a recent study, we demonstrated the feasibility of electroporating CRISPR-Cas9 mRNA and ribonucleoprotein (RNPs) into primary mouse and human hepatocytes and showed high levels of gene-editing activity.^33,34^ Electroporation-mediated delivery of CRISPR components performed ex vivo as part of cell therapy is potentially safer than systemic delivery because gene editing only occurs in the intended target cell type. Ex vivo gene editing for liver disease is associated with additional processing steps: cell isolation from the host-resected liver, gene editing, and transplantation to replace diseased hepatocytes with healthy ones (graphical abstract). Ex vivo gene editing has been demonstrated in a *Fah*^−/−^ mouse model of HT1 using viral vectors^35–37^ but not using nonviral delivery approaches.

In this study, we demonstrate a successful cell therapy approach to reprogram metabolic pathways by electroporating CRISPR-Cas9 mRNA and RNPs to disrupt *Hpd*, a therapeutic gene, in primary hepatocytes ex vivo, followed by transplantation to treat HT1 in a *Fah*^−/−^ mouse model as proof of principle. We developed a cytokine recovery medium that was transiently incubated with electroporated hepatocytes and found that this medium was a critical step in obtaining high levels of liver repopulation. Our optimized gene-editing protocol enabled primary hepatocytes from *Fah*^−/−^ mice to repopulate the liver, protect against acute liver failure, and reverse the HT1 disease phenotype in transplanted recipients.

## METHODS

### Animals and animal care

All mice received humane care in compliance with the Institutional Animal Care and Use Committee regulations of Clemson University. To establish the hepatocyte transplantation protocol, we isolated hepatocytes from C57BL/6-Tg(CAG-EGFP)1Osb/J mice (GFP mice). Thereafter, wild-type C57BL/6J mice maintained on a regular chow diet (Teklad, 2018) were used to isolate healthy donor hepatocytes. C57BL/6J *Fah*∆exon5 (*Fah*^−/−^) mice containing a 105 bp deletion in exon 5 of the *Fah* were a generous gift from Dr Markus Grompe at the Oregon Health and Science University (Portland, OR). The *Fah*^−/−^ mice were used to isolate diseased hepatocytes for gene editing and as recipients of hepatocyte transplantation to assess our cell therapy approach for HT1. *Fah*^−/−^ mice were maintained on a high-energy chow diet (PicoLab, 5LJ5) and placed on drinking water containing 8 mg/mL NTBC (Ark Pharm). Transplanted *Fah*^−/−^ mice were imaged using an IVIS Lumina XR small animal imaging system (Caliper Life Sciences).

### Cytokine recovery media for electroporated hepatocytes

We developed a recovery medium and added it to hepatocytes after electroporation to increase cell viability. Cytokines were ordered from PeproTech and reconstituted to prepare the following stock solutions: 3 mM CHIR-99021 (glycogen synthase kinase [GSK] 3 inhibitor) in DMSO, 5 mM A83-01 (activin receptor–like kinase 5 inhibitor) in DMSO, 500 μg/mL human EGF in PBS with 0.1% (w/v) bovine serum albumin, 100 μg/mL human HGF in PBS with 0.1% (w/v) bovine serum albumin, 100 mM Y-27632 (rho-associated, coiled-coil–containing protein kinase [ROCK] inhibitor) in sterile dH_2_O, and 500 mM *N*-acetyl-l-cysteine (NAC) in sterile dH_2_O. At 24 hours before electroporation, cytokine stock solutions were thawed at room temperature and added to ice-cold HMX media (DMEM high glucose with GlutaMAX, 10% fetal bovine serum, 100 U/mL penicillin, 0.1 mg/mL streptomycin, and 10 mM HEPES) in the following (v/v) amounts: 1.0% 3 mM CHIR-99021, 0.2% 5 mM A83-01, 0.1% 500 μg/mL human EGF, 0.5% 100 μg/mL human HGF, 0.5% 100 mM Y-27632, and 2.5% 500 mM NAC.

### Isolation of primary hepatocytes and electroporation

Hepatocytes were isolated from male wild-type C57BL/6J, GFP, or *Fah*^*−*/*−*^ mice 8–10 weeks old using a 3-step perfusion procedure described.^34^ Hepatocyte viability was quantified by trypan blue staining using an automated cell counter. Hepatocytes isolated with a yield of 10–40×10^6^ cells and >80% viability were used in electroporation experiments immediately after perfusion and washing steps. Freshly isolated primary mouse hepatocytes were electroporated with a 2b Nucleofector device (Lonza) using the program T-028 as described.^33^

Briefly, Hepa 1-6 cells (American Type Culture Collection) were cultured in DMEM (Gibco) supplemented with 10% fetal bovine serum, 4 mM l-glutamine, and 1× antibiotic-antimycotic at 37°C in a humidified incubator with 5% CO_2_ and ambient oxygen levels. Electroporation in Hepa 1-6 cells was carried out with a 4D Nucleofector X Unit (Lonza) using the CM-138 program as described in Rathbone et al.^33^

### Hepatocyte transplantation through intrasplenic injection

For each transplantation, 500,000 electroporated or untreated hepatocytes were resuspended in 120 µL of ice-cold HMX medium and, within 2 hours after electroporation, intrasplenically injected into male *Fah*^*−*/*−*^ recipient mice, 6–10 weeks old. Recipient mice were withdrawn from NTBC 3 days before transplantation to stimulate engraftment. Isoflurane was used for anesthesia induction and maintenance in the recipient mice. A small 5–10 mm vertical incision in the upper left side of the abdomen was used to visualize the spleen. Hepatocytes were injected using a 30-gauge syringe into the inferior tip of the spleen. Next, the peritoneum was sutured and the skin was closed using clips.

After transplantation, recipient mice remained off NTBC water to activate the expansion of transplanted wild-type or *Hpd*-deficient hepatocytes in the liver. During the NTBC withdrawal period, drinking water was supplemented with 32.5 g/L dextrose, and the mice were weighed every 2–3 days. Once a 15%–20% weight decrease was observed, mice were immediately switched to water supplemented with 8 mg/L NTBC and 35.7 g/L dextrose to inhibit toxicity. Once the initial weights were restored, the mice were switched to water supplemented with dextrose. The cycle of placing recipient mice on and off NTBC water continued until the weights stabilized independently of NTBC.

### Quantification of gene-editing efficiency

Gene-editing efficiency was quantified using previously described methods.^33^ The PCR primers used for the *Hpd* target site are listed in Supplemental Table S1, http://links.lww.com/HC9/A847. The editing efficiency in hepatocytes was quantified by dividing the indels by 0.6 as a correction factor to account for hepatocytes making up 60% of the total liver DNA.^38^

### Histology and immunohistochemistry

Liver lobes were cut into ~3-mm-thick sections, and tissue samples were fixed in 10% neutral-buffered formalin (Thermo Fisher Scientific). Standard protocols were followed for hematoxylin and eosin (H&E) staining. Masson’s trichrome staining kit (Abcam) was used for tissue sections following the manufacturer’s protocol. For the Fah immunohistochemistry (IHC) staining, described methods were used^39^ with the following primary antibodies: mouse anti-Fah^40^ (1:600) or mouse anti-Hpd (Santa Cruz, 1:100). The percentage of engraftment was quantified from IHC images of the liver stained against Fah or Hpd using ImageJ software (Rasband, W.S., ImageJ, US National Institutes of Health, https://imagej.nih.gov/ij/).

### Metabolic analysis

Blood was collected from all experimental mice by cardiac puncture, and the serum was separated by centrifugation. Serum samples were analyzed for tyrosine and phenylalanine concentrations using tandem mass spectrometry and chromatography (Mayo Clinic). The liver and kidney biochemical markers, lipid levels, and glucose levels were analyzed in the serum using a custom chemistry panel (Idexx BioAnalytics). The Hpd concentration was measured in Hepa 1-6 cell lysates and liver tissue homogenates from experimental mice using a Mouse Hpd ELISA kit (MyBioSource) according to the manufacturer’s instructions.

### Statistical analysis

All statistical analyses on gene editing, serum biochemical markers, and amino acid data were performed using GraphPad Prism software. Statistical significance was set at *p*<0.05. Experimental differences between multiple groups were compared using one-way ANOVA, followed by Tukey’s correction for multiple comparisons. For all statistical analyses, *p<0.05, **p<0.01, ***p<0.001, and ****p<0.0001. All error bars indicate the SEM.

## RESULTS

### Cytokine recovery medium improves engraftment efficiency

We previously designed and validated sgRNA-targeting *Hpd* that provides a high frequency of on-target frameshift mutations when delivered as RNPs (76.2%) in freshly isolated primary mouse hepatocytes.^33^ First, we validated that *Hpd*-targeting CRISPR-Cas9 knocked down Hpd expression. In Hepa 1-6 cells electroporated with *Hpd*-Cas9 RNPs, a significant reduction in Hpd was observed (Supplemental Figure S1, http://links.lww.com/HC9/A847) compared to untreated controls (mean: 11.9 and 24.2 ng/mL; respectively, *p*=0.0242). Next, we established the hepatocyte transplantation procedure in *Fah*^*−*/*−*^ mice, a model of HT1, by splenically injecting primary hepatocytes freshly isolated from healthy GFP mice. After 1 cycle off-NTBC and then on-NTBC, the transplanted mice remained stable off NTBC starting 22 days after transplantation (Supplemental Figure S2, http://links.lww.com/HC9/A847). The engraftment was verified by in vivo fluorescence imaging (Supplemental Figure S3, http://links.lww.com/HC9/A847) and IHC staining against Fah in liver tissue sections (Supplemental Figure S4, http://links.lww.com/HC9/A847).

**FIGURE 1:**
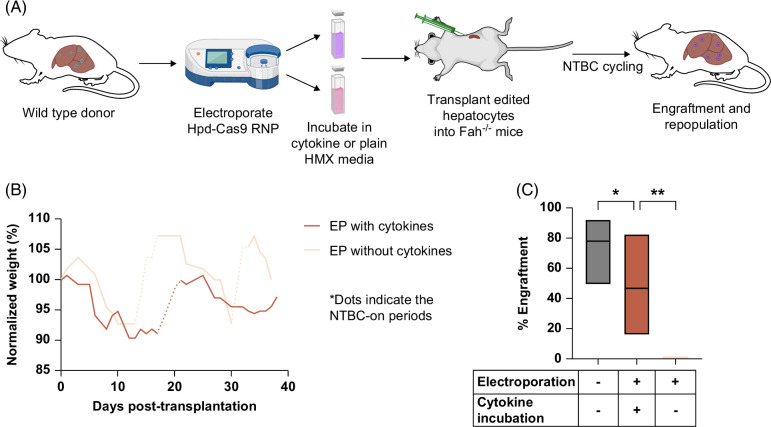
Transplantation of cytokine-treated hepatocytes electroporated with *Hpd*-targeting CRISPR-Cas9 RNPs. (A) Schematic of the experimental setup. (B) Mean normalized weight data for recipient *Fah*^−/−^ mice (n=5) after transplantation with wild-type hepatocytes EP with *Hpd*-Cas9 RNPs and incubated with cytokine recovery media or plain HMX media. The dotted lines indicate the NTBC-on periods and the solid lines represent the NTBC-off periods. (C) Percent liver engraftment estimated using IHC staining against Fah (lines inside the box plot represent mean levels, and the lower and upper bars represent the minimum and the maximum values). Levels of significance *p<0.05, **p<0.01 (one-way ANOVA with Tukey multiple comparison). Abbreviations: EP, electroporated; NTBC, 2-(2-nitro-4-trifluoro-methylbenzyol)-1,3 cyclohexanedione; RNP, ribonucleoprotein.

We next investigated the effects of electroporation on the capacity of hepatocytes to engraft in the liver. Hepatocytes were isolated from wild-type C57BL/6J mice and electroporated with *Hpd*-targeting Cas9 RNP. After electroporation and 15-minute incubation on ice, the cell suspension was split into 2 treatment groups: (1) an additional 15-minute incubation in cytokine recovery medium or (2) an additional 15 minute-incubation in plain HMX medium (Figure 1A). The cytokine recovery medium was prepared with cytokines to inhibit apoptosis: ROCK inhibitor (Y-27632), GSK3 inhibitor (CHIR 99021), activin receptor–like kinase 5 inhibitor (A-83-01), NAC, human EGF, and human HGF. We included ROCK inhibitor because it blocks apoptosis in rat hepatocytes.^41^ The GSK3 inhibitor was included because GSK3 is involved in diverse signaling pathways governing cell death and survival, including promoting intrinsic apoptotic pathways upon cell damage.^42^ Another important apoptotic pathway relies on TGFβ induction. Godoy et al^43^ demonstrated that stimulating hepatocytes with an activin receptor–like kinase 5 inhibitor abolished TGF-β–induced apoptosis. We added EGF to the cytokine recovery medium because it improves the viability and biochemical integrity of plated hepatocytes.^44^ HGF injection into the portal vein results in hepatocyte proliferation and liver enlargement in rats and mice,^45^ indicating its importance for liver regeneration. Studies show that NAC has hepatoprotective and anti-inflammatory effects, protecting hepatocytes from ischemic damage and decreasing apoptosis.^46,47^ After brief incubation in HMX medium with or without the recovery-stimulating cytokines, electroporated hepatocytes were washed, resuspended in ice-cold plain HMX media, and intrasplenically injected into *Fah*^−/−^ recipients. At the endpoint, all mice transplanted with electroporated hepatocytes that were transiently incubated in cytokine recovery medium had weights that stabilized independently of NTBC. In contrast, the electroporated hepatocytes incubated in HMX media without cytokines required additional NTBC administration and displayed a consistent weight reduction (Supplemental Figure S5, http://links.lww.com/HC9/A847) compared to the NTBC-on levels by the endpoint (Figure 1B). This indicated that the recipients did not achieve NTBC independence due to insufficient engraftment by donor hepatocytes. The engraftment levels were quantified by IHC staining of Fah in liver tissue sections (Supplemental Figure S6, http://links.lww.com/HC9/A847). Mice transplanted with electroporated hepatocytes transiently incubated in cytokine recovery medium showed significantly higher engraftment levels (Figure 1C) compared to electroporated hepatocytes incubated in plain HMX medium without cytokines (mean: 46.8% and 0.83%, respectively; *p*=0.0025). In conclusion, the results indicate that brief incubation in the cytokine recovery medium was critical for retaining hepatocyte viability and engraftment potential after electroporation. Therefore, we incorporated the 15-minute cytokine recovery medium incubation step into our electroporation procedure for all subsequent transplantation experiments.

### Electroporated hepatocytes from wild-type C57BL/6J mice correct the HT1 disease phenotype

**FIGURE 2:**
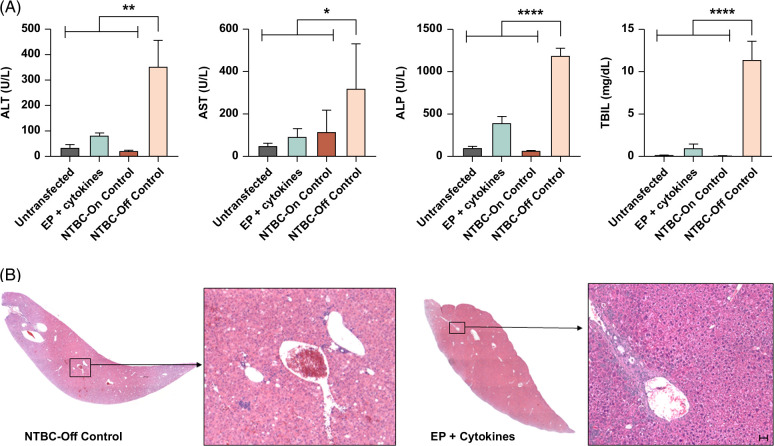
Electroporated hepatocytes isolated from wild-type donor mice protect against liver failure in Fah^−/−^ recipient mice. (A) Levels of liver biomarkers in serum: ALP, ALT, AST, and TBIL, respectively, for recipients transplanted with untransfected wild-type hepatocytes or EP and incubated with cytokine recovery media. Untransplanted mice maintained on NTBC or kept off NTBC were used as controls. Bars represent the mean (n=5), and error bars represent SEM. Levels of significance *p<0.05, **p<0.01, and ****p<0.0001 (one-way ANOVA with Tukey multiple comparison). (B) Representative H&E-stained histological images of the liver for recipient mice transplanted with Cas9 RNP and cytokine-treated hepatocytes compared with *Fah*^−/−^ controls kept off NTBC. The scale bar represents 50 μm. Abbreviations: ALP, alkaline phosphatase; ALT, alanine transaminase; AST, aspartate transaminase; EP, electroporated; NTBC, 2-(2-nitro-4-trifluoro-methylbenzyol)-1,3 cyclohexanedione; RNP, ribonucleoprotein; TBIL, total bilirubin.

Next, we assessed whether hepatocytes isolated from wild-type C57BL/6J mice retained their functionality after electroporation to rescue *Fah*^−/−^ mice. After termination, we collected serum from recipient mice and measured the biochemical enzymes associated with liver function. Biochemical results (Figure 2A) revealed that total bilirubin (TBIL), alanine transaminase, aspartate transaminase, and alkaline phosphatase levels decreased in electroporated hepatocytes that were briefly incubated in cytokine recovery medium compared to untreated *Fah*^−/−^ mice kept off NTBC (NTBC-off controls). The H&E (Figure 2B) and trichrome staining (Supplemental Figure S7, http://links.lww.com/HC9/A847) in liver tissues of mice transplanted with electroporated hepatocytes incubated with cytokine recovery medium revealed no signs of major pathology or fibrosis. In contrast, steatosis was observed in the NTBC-off controls (Supplemental Table S2, http://links.lww.com/HC9/A847). These results suggest our electroporation procedure does not adversely affect hepatocyte functionality in vivo following liver repopulation.

### Gene-edited hepatocytes from diseased mice engraft and repopulate the liver in *Fah*^−/−^ recipients

**FIGURE 3:**
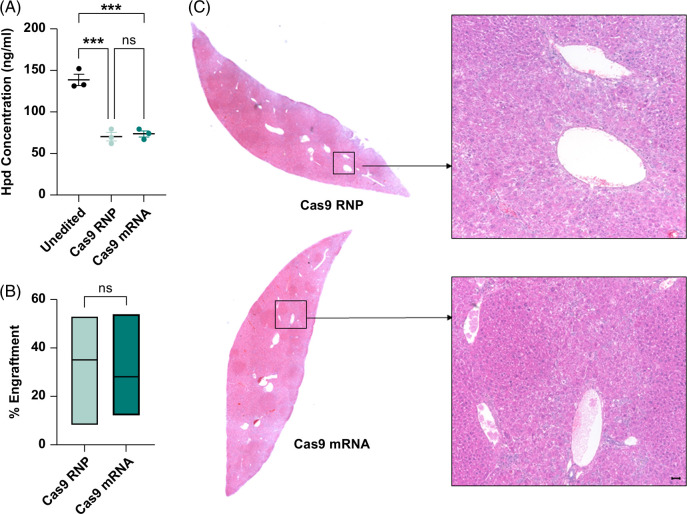
Diseased hepatocytes electroporated with *Hpd*-Cas9 mRNA and RNPs engraft in *Fah*^−/−^ recipient mice. (A) Quantitative data for Hpd protein levels measured by ELISA assay (n=5, error bars represent SEM, and each dot represents a different sample). Level of significance ***p<0.001 (one-way ANOVA with Tukey multiple comparison). (B) Percent engraftment in transplanted recipients (n=5) estimated by immunohistochemical staining against Hpd (lines inside the box plot represent mean levels, and the lower and upper bars represent the minimum and the maximum values). (C) Representative H&E-stained histology liver images from recipient mice transplanted with hepatocytes electroporated with *Hpd*-Cas9 RNP and mRNA. The scale bar represents 50 μm. Abbreviations: H&E, hematoxylin and eosin; RNP, ribonucleoprotein.

We established proof-of-principle application of our electroporation procedure for ex vivo gene editing to correct HT1, whereby hepatocytes from *Fah*^−/−^-diseased mice were electroporated with *Hpd*-targeting Cas9 RNP or mRNA and subsequently transplanted into *Fah*^−/−^ recipient mice through splenic injection. First, we validated that *Hpd*-sgRNA knocked down Hpd protein using an ELISA assay performed on liver tissue samples at 100 days after transplantation (Figure 3A). Compared to untreated *Fah*^−/−^ control mice, transplanted mice receiving hepatocytes electroporated with *Hpd*-Cas9 mRNA (mean: 73 and 139 ng/mL, respectively; *p*=0.0002) and RNP (mean: 70 and 139 ng/mL, respectively; *p*=0.0002) had significantly reduced Hpd levels. We observed no significant difference in Hpd levels between mice transplanted with *Hpd*-Cas9 RNP or mRNA (Figure 3A). The engraftment of gene-edited hepatocytes was quantified using IHC images of the liver tissue from *Fah*^−/−^ recipient mice stained for Hpd (Supplemental Figure S8, http://links.lww.com/HC9/A847). The mice transplanted with Cas9 RNP-treated hepatocytes showed an average of 35% engraftment, while the Cas9 mRNA transplanted mice showed 28% engraftment by Hpd-deficient-edited hepatocytes (Figure 3B). H&E-stained images revealed no major pathology in mice transplanted with *Hpd*-Cas9 mRNA or RNP (Figure 3C; Supplemental Table S2, http://links.lww.com/HC9/A847) and were consistent with the gross liver images that showed improved physiology and tumor pathology compared to controls at 100 days after transplantation (Supplemental Figure S9, http://links.lww.com/HC9/A847).

### Transplantation of ex vivo gene-edited hepatocytes corrects HT1 disease in *Fah*^−/−^ mice

**FIGURE 4:**
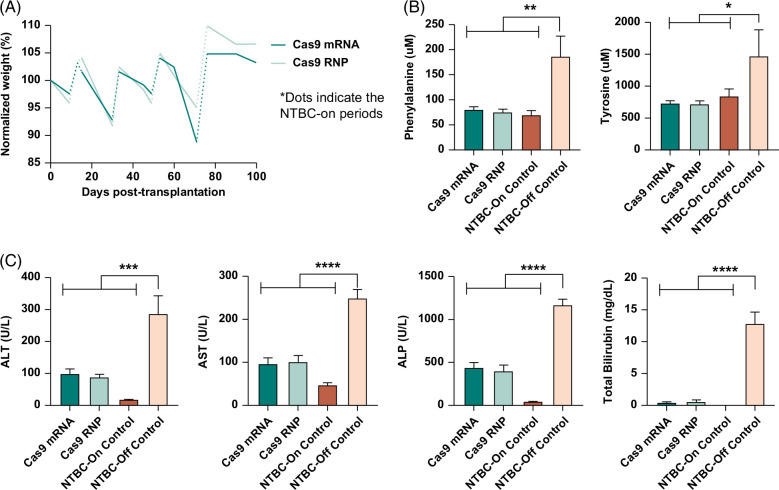
Correction of HT1 phenotype in *Fah*^−/−^ recipient mice transplanted with diseased hepatocytes electroporated with *Hpd*-Cas9 mRNA and RNPs. (A) Progressive weight data in transplanted *Fah*^−/−^ recipients on-NTBC and off-NTBC (n=5). The dotted lines indicate the NTBC-on periods and the solid lines represent the NTBC-off periods. (B) Mean phenylalanine and tyrosine levels (n=5) with error bars representing the SEM. (C) Biochemical markers of liver function were measured in serum for transplanted recipients and control mice (n=5) with error bars representing the SEM. Levels of significance *p<0.05, **p<0.01, ***p<0.001, and ****p<0.0001 (one-way ANOVA with Tukey multiple comparison). Abbreviations: ALP, alkaline phosphatase; ALT, alanine transaminase; AST, aspartate transaminase; NTBC, 2-(2-nitro-4-trifluoro-methylbenzyol)-1,3 cyclohexanedione; RNP, ribonucleoprotein.

Next, we investigated the therapeutic potential of our ex vivo gene-editing approach involving electroporation of *Hpd*-Cas9 mRNA or RNP into *Fah*^−/−^ diseased hepatocytes, followed by transplantation into *Fah*^−/−^ recipient mice. The weight of the transplanted recipients was closely monitored (Supplemental Figure S10, http://links.lww.com/HC9/A847) and stabilized at 100 days after transplantation (Figure 4A) with a 100% survival rate. A comprehensive biochemical analysis was performed on the serum collected from transplanted recipients at the endpoint to measure the levels of phenylalanine and tyrosine (Figure 4B) as well as biochemical markers of liver injury (Figure 4C). Compared to NTBC-off control mice, phenylalanine levels decreased significantly for both Cas9 RNP (mean: 75.8 and 187 μM, respectively; *p*=0.0006) and mRNA-transplanted mice (mean: 81.2 and 187 μM, respectively; *p*=0.0026). In addition, compared to NTBC-off control mice, tyrosine levels were reduced in Cas9 RNP (mean: 719 and 1474 μM; *p*=0.0256, respectively) and Cas9 mRNA–treated mice (mean: 730 and 1474 μM; *p*=0.0514, respectively). We observed a significant reduction in liver enzymes and TBIL levels in mice transplanted with Cas9 mRNA and RNP-treated hepatocytes compared to NTBC-off control mice. There was no significant difference in the Cas9 RNP and mRNA-treated mice for all biochemical markers of liver function. These results indicate that hepatocytes electroporated with *Hpd*-CRISPR-Cas9 using our optimized protocol can engraft and phenotypically correct HT1 disease in *Fah*^−/−^ mice.

### Engraftment efficiency improves by increasing the number of viable hepatocytes transplanted

**FIGURE 5:**
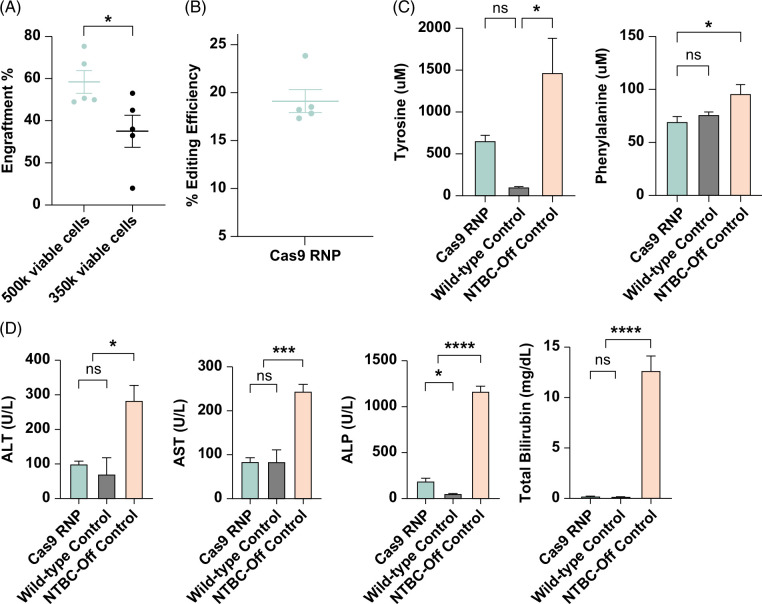
Establishing the dose by the number of viable hepatocytes after electroporation improves engraftment. (A) Percent engraftment estimated by IHC staining against Hpd (error bars represent SEM, and each dot represents a different mouse) in mice transplanted with 350,000 or 500,000 viable cells after electroporating *Hpd*-Cas9 RNP. (B) The percent editing efficiency estimated by TIDE analysis of on-target indels in gDNA isolated from homogenized liver from recipients transplanted with 500,000 viable hepatocytes after electroporation. (C) Phenylalanine and tyrosine levels and (D) biochemical markers in serum from recipients transplanted with 500,000 viable cells. The controls consisted of untreated wild-type C57BL/6J mice and *Fah*^*−/−*^ mice kept off NTBC. Horizontal lines or bars represent mean levels (n=5), and error bars represent the SEM. Levels of significance *p<0.05, ***p<0.001, and ****p<0.0001 (one-way ANOVA with Tukey multiple comparison). Abbreviations: ALP, alkaline phosphatase; ALT, alanine transaminase; AST, aspartate transaminase; IHC, immunohistochemistry; NTBC, 2-(2-nitro-4-trifluoro-methylbenzyol)-1,3 cyclohexanedione; RNP, ribonucleoprotein.

We hypothesized that increasing the number of transplanted electroporated hepatocytes would improve the engraftment efficiency. To test our hypothesis, we splenically injected 500,000 viable *Fah*^−/−^ hepatocytes immediately after electroporating *Hpd*-Cas9 RNP. In our previous experiment, we splenically injected 500,000 electroporated hepatocytes, including 350,000 viable cells transplanted per recipient mouse (Supplemental Table S3, http://links.lww.com/HC9/A847). At 109 days after transplantation, recipient mice were sacrificed and the engraftment was analyzed using IHC staining against Hpd (Supplemental Figure S11, http://links.lww.com/HC9/A847). We observed an increase in mean engraftment from 35% to 58% when the number of viable electroporated hepatocytes transplanted was increased from 350,000 to 500,000 (Figure 5A). The on-target gene-editing efficiency was 19% in genomic DNA isolated from digested liver harvested from transplanted recipient mice (Figure 5B). In mice transplanted with Cas9 RNP–treated hepatocytes, we observed a significant decrease in the levels of phenylalanine and tyrosine and biochemical liver markers compared to NTBC-off control mice (Figures 5C, D). There was no statistically significant difference between healthy age-matched wild-type control and Cas9 RNP–transplanted recipients in alanine transaminase, aspartate transaminase, TBIL, and phenylalanine levels. In addition, mice transplanted with Cas9 RNP–treated hepatocytes showed significant improvements in biochemical markers of renal function, blood glucose levels, and cholesterol levels compared to diseased NTBC-off controls (Supplemental Figure S12, http://links.lww.com/HC9/A847). The H&E and trichrome staining in the liver sections of Cas9 RNP–treated mice revealed no signs of major pathology or fibrosis (Supplemental Figure S13, http://links.lww.com/HC9/A847). The absence of Fah in HT1 disease induces oxidative damage and stress responses linked to the Kelch-like ECH-associated protein 1 (Keap1)/nuclear factor erythroid 2–related factor 2 (Nrf2) signaling pathway.^39^ Therefore, we evaluated the effects of our gene-editing treatment on oxidative stress by analyzing the RNA expression of proteins related to the oxidative stress response. We observed a decrease in total *Nrf2* (*Nfe212*) mRNA expression in Cas9 RNP–treated mice compared to diseased NTBC-off controls, and there was no significant difference in *Nrf2* expression in Cas9 RNP–treated mice compared to wild-type C57BL/6J controls, indicating that knockdown of *Hpd* in *Fah*^−*/*−^ hepatocytes normalized the *Nrf2* expression. In addition, we observed a significant reduction in gene expression of Nrf2 targets and a concomitant decrease in HCC markers *Mat2a* (methionine adenosyltransferase 2A), *Afp*, *Gpc3* (glypican 3), *c-Myc* in Cas9-treated mice compared to NTBC-off–diseased mice (Supplemental Figure S14, http://links.lww.com/HC9/A847). The results demonstrate that recipients tolerated the higher dose of transplanted hepatocytes, affirming the effectiveness of our gene-editing approach in rescuing *Fah*^−*/*−^ mice from liver and renal dysfunction, as well as hypoglycemia. Furthermore, our approach mitigated oxidative stress linked to Fah deficiency in HT1 and inhibited the induction of HCC markers.

## DISCUSSION

Our study represents a significant advancement in HT1 therapy and is pivotal for the progress of ex vivo liver cell therapy in a broader context. Utilizing the well-established *Fah*^−/−^ mouse model, we demonstrated the feasibility of our ex vivo gene-editing approach, serving as a proof-of-principle model for IMDs of the liver. We show the feasibility of ex vivo electroporation of primary hepatocytes, followed by transplantation, to successfully rescue the HT1 disease phenotype. Our gene-editing approach for metabolic pathway reprogramming^48,49^ has the potential to benefit not only HT1 but also other IMDs.

We demonstrated our ex vivo gene therapy approach in *Fah*^−/−^ mice because *Hpd*-deficient hepatocytes have a natural selective advantage over native mutant hepatocytes to replicate and rescue the HT1 phenotype.^50^ We observed up to 75% engraftment when diseased hepatocytes electroporated with *Hpd*-CRISPR-Cas9 were transplanted into *Fah*^−/−^ mice (Figure 5A). Hepatocytes electroporated with Cas9 RNP provided slightly higher engraftment levels than those electroporated with mRNA, although this was not statistically significant (Figure 3B). This result is consistent with the findings in our prior study that *Hpd*-CRISPR-Cas9 delivered as an RNP provides higher levels of on-target indels than mRNA.^33^ These results demonstrate that electroporation-mediated delivery of CRISPR-Cas9 into hepatocytes is efficient and yields viable cells that can engraft and repopulate the liver of *Fah*^−/−^ mice.

The discrepancy observed between the engraftment rates of edited hepatocytes (Figure 5A) and the efficiency of generating indels (Figure 5B) can be explained by considering the diverse nuclear content within the liver. The withdrawal of NTBC stimulates the proliferation of hepatocytes lacking Hpd, leading to the regeneration of hepatocyte populations. Hepatocytes with higher nuclear content, particularly polyploid ones, tend to have a reduced capacity for proliferation compared to their diploid counterparts.^51^ Thus, it is crucial to consider that the presence of polyploid native hepatocytes and unedited hepatocytes in liver tissue can influence the overall efficiency of gene editing, as indicated by the indel efficiency. The impact of these nonedited polyploid hepatocytes can dilute the indels in gDNA extracted from bulk homogenized tissue.

Our metabolic analyses showed that electroporation of *Hpd*-Cas9 RNP and mRNA into *Fah*^−/−^ hepatocytes followed by transplantation into diseased mice reversed the levels of liver injury biomarkers, including alanine transaminase, aspartate transaminase, alkaline phosphatase, and TBIL (Figure 4C). The glucose levels in Cas9 RNP–treated mice were significantly higher than the diseased NTBC-off controls (mean: 179.4 and 5.5 mg/dL; *p* = 0.0006), indicating correction in the hypoglycemia phenotype (Supplemental Figure S12, http://links.lww.com/HC9/A847) characteristic of *Fah*^−*/*−^ mice off NTBC.^52^ In addition, transplantation of Cas9 RNP–treated hepatocytes resulted in the normalization of biochemical markers of renal injury and cholesterol levels compared with NTBC-off diseased controls (Supplemental Figure S12, http://links.lww.com/HC9/A847). We observed no statistical difference between Cas9 RNP and mRNA-treated mice in biochemical markers and amino acid levels, which normalized within 2.5 months following transplantation (Figure 4). Furthermore, *Fah*^−/−^ mice transplanted with *Hpd*-Cas9 RNP and mRNA stabilized their weights independently of NTBC. The images of the H&E-stained sections and gross livers consistently showed that the *Fah*^−/−^ mice transplanted with gene-edited hepatocytes had healthier physiology and tumor improvement than the untreated NTBC-off controls (Supplemental Figure S9, http://links.lww.com/HC9/A847). Taken together, these results provide evidence of liver repopulation by transplanted hepatocytes electroporated with *Hpd*-targeting CRISPR-Cas9 to correct HT1 disease.

Previous ex vivo gene-editing studies to treat HT1 used viral vectors to deliver sgRNA and Cas9 in vivo or ex vivo into primary hepatocytes followed by transplantation.^36,37,48,53^ VanLith and colleagues demonstrated gene correction of a single-point mutation in exon 8 of the *Fah* using AAV vectors carrying *Fah*-aiming CRISPR-Cas9 and a donor template. Although repopulation by gene-corrected hepatocytes was not directly quantified, they observed 12% targeted gene-editing efficiency.^36^ In all previous studies, disease indicators, including liver failure enzymes, were reduced to normal levels, which aligns with our findings. Viral delivery approaches have significant drawbacks including risks of immune responses and potential insertional mutagenesis. AAV integration frequencies as high as 1%–3% have been observed in human hepatocytes.^54^ In addition, ex vivo delivery using AAVs is associated with loss of cell viability and functionality due to excessive culturing steps during the viral transduction procedure.^36,55^ In contrast, we show that electroporation facilitates rapid and efficient delivery of CRISPR-Cas9 as mRNA and RNPs, which exist for short periods of time, in hepatocytes while in suspension as a potentially safer alternative to viral methods.

The impacts of electroporation on generating models have been shown in a study by Zabulica et al,^56^ involving the use of primary human hepatocytes obtained from patients that were subsequently transplanted into *Fah*^*−/−*^, *Rag2*^*−/−*^, and *Il2rg*^*−/−*^ mice on the NOD-strain background to create a humanized chimeric model of ornithine transcarbamylase deficiency. A major limitation of this study is that the *Fah*^*−/−*^, *Rag2*^*−/−*^, and *Il2rg*^*−/−*^ mice on the NOD-strain background mice have severely compromised immune systems, which creates a permissive environment for engraftment and does not accurately represent the interaction of human hepatocytes with a functional immune system. A critical challenge in hepatocyte transplantation is that the immune system acts as a barrier to successful engraftment. In the presence of functional immune cells, more than 70% of donor hepatocytes are cleared within 2–24 hours after transplantation, limiting their survival and integration into the liver parenchyma.^57^ Furthermore, the recipient *Fah*^*−/−*^, *Rag2*^*−/−*^, and *Il2rg*^*−/−*^ mice on the NOD-strain background mice used in Zabulica et al^56^ do not represent a mouse model of ornithine transcarbamylase deficiency; therefore, the study did not demonstrate correction of the disease phenotype in the host. In contrast, our study used HT1 mice with functional immune systems as recipients for engraftment of gene-edited hepatocytes to better replicate the clinical application of our cell therapy approach. We introduced a cytokine recovery medium to increase engraftment after electroporation. Despite the challenges posed by the immune system in the *Fah*^−/−^ mice, we successfully demonstrated engraftment with correction, indicating its therapeutic potential in treating HT1.

The RNA expression analysis indicates that our ex vivo gene-editing approaches using *Hpd*-CRISPR-Cas9 ameliorated oxidative stress and protected against the induction of HCC markers in *Fah*^−*/*−^ mice. Consistent with our findings, the study conducted by Gu and colleagues showed that alpha-fetoprotein expression was significantly higher in *FAH*^−*/*−^-diseased pigs than in double mutant *FAH*^−*/*−^ */HPD*^−*/*−^ or WT pigs. Homozygous null mutations in Hpd were shown to lower oxidative stress–related gene expression and alpha-fetoprotein induction associated with Fah deficiency in an HT1 pig model.^39^ Long-term studies in albino homozygous C^14CoS^ Fah-deficient mice indicate that therapeutic deletion of *Hpd* in double mutant *Fah*^−*/*−^ */Hpd*^−*/*−^ mice protects against liver disease and hepatocyte apoptosis.^58^ In contrast, *Fah*^−*/*−^ mice treated with NTBC develop tumors in the liver because of downstream toxic metabolites, which remain elevated as a result of incomplete blockage of Hpd by the drug.^58,59^ Our study showed that electroporation of *Hpd*-CRISPR-Cas9 into hepatocytes did not result in any visible tumors in *Fah*^−*/*−^ mice. In addition, our cell-based ex vivo approach is suitable for metabolic liver diseases, in which only a small fraction of native hepatocytes must be replaced by edited cells. Hence, HT1 is rather an exception, but there are also other IMDs in which corrected hepatocytes have a selective growth advantage over native hepatocytes.^60,61^

One limitation of electroporation is its toxicity to cells. Even low-electric-field-strength pulses can lead to cell injury, including membrane damage, ATP depletion, and increased reactive oxygen species, leading to cell death.^62,63^ To achieve successful engraftment in the liver, it is essential to minimize cell death after electroporation in hepatocytes. Apoptosis has been identified as a major pathway of cell death after electroporation.^63,64^ To overcome electroporation-induced apoptosis, we prepared a cytokine recovery medium containing antiapoptotic factors to increase cell viability and functionality. Our results showed a significant increase in liver engraftment when primary hepatocytes were transiently incubated in a cytokine recovery medium immediately after electroporation (Figure 1C). The cytokine recovery media contains antiapoptotic factors that are not specific to hepatocytes and can potentially enhance viability and functionality postelectroporation in other cell types.

In summary, our results demonstrated the efficacy and safety of an electroporation-mediated ex vivo protocol for therapeutic CRISPR-Cas9 gene editing in a mouse model of HT1. Our work shows the impacts of electroporation combined with hepatocyte transplantation as a potential autologous cell therapy for IMDs of the liver.

## Supplementary Material

SUPPLEMENTARY MATERIAL
