# Supplementary material for: Ex vivo gene editing and cell therapy for hereditary tyrosinemia type 1
Source: Hepatol Commun. 2024 Apr 26;8(5):e0424. doi: 10.1097/HC9.0000000000000424 (PMC12333821; doi:10.1097/HC9.0000000000000424)
Supplement: SUPPLEMENTARY MATERIAL [file hc9-8-e0424-s001.pdf]

CURATIVE EX VIVO GENE EDITING AND CELL THERAPY FOR HEREDITARY TYROSINEMIA TYPE 1

Ilayda Ates[1], Callie Stuart[1], Tanner Rathbone[1], Mercedes Barzi[2], Gordon He[2], Angela M. Major[3], Vijay Shankar[4,5], Rachel A Lyman[4,5], Sidney S. Angner[4,5], Trudy F C Mackay[4,5], Shanthi Srinivasan[6], Alton Brad Farris[7], Karl-Dimiter Bissig [2,8,9,10,11], Renee N. Cottle[1,*]

[1] Department of Bioengineering, Clemson University, Clemson, SC 29634, USA

[2] Alice and Y. T. Chen Center for Genetics and Genomics, Division of Medical Genetics, Department of Pediatrics, Duke University School of Medicine, Durham, NC 27710, USA

[3] Department of Pathology, Texas Children's Hospital, Houston, TX 77030, USA

[4] Department of Biochemistry and Genetics, Clemson University, Clemson, SC 29634, USA

[5] Center for Human Genetics, Clemson University, Greenwood, SC, 29646, USA.

[6] Digestive Diseases Division, Department of Medicine, Emory University School of Medicine, Atlanta, GA 30322, USA

[7] Department of Pathology and Laboratory Medicine, Emory University School of Medicine, Atlanta, GA 30322, USA

[8]Division of Gastroenterology, Department of Medicine, Duke University Medical Center, Durham, NC 27710, USA

[9]Department of Biomedical Engineering (BME) at the Duke University Pratt School of Engineering, Durham, NC 27710, USA

[10]Duke Cancer Center, Duke University Medical Center, Durham, NC 27710, USA

[11]Department of Pharmacology and Cancer Biology, Duke University Medical Center, Durham, NC 27710, USA

Table of Contents

Supplementary Methods

Hepatocyte electroporation and RNA expression analysis

Supplementary Figures

Supplementary Figure 1. Hpd concentration after electroporating Hpd-Cas9 into Hepa 1-6 cells.

Supplementary Figure 2. Weight data of Fah-/- mice transplanted with unedited wild-type hepatocytes.

Supplementary Figure 3. Representative IVIS images of Fah-/- recipient mice transplanted with wild-type GFP hepatocytes.

Supplementary Figure 4. IHC staining against Fah in Fah-/- mice transplanted with wild-type GFP hepatocytes.

Supplementary Figure 5. Progressive weight data of Fah-/- mice transplanted with hepatocytes electroporated with Hpd-Cas9 RNP with or without the cytokine recovery media.

Supplementary Figure 6. IHC staining against Fah in liver section from Fah-/- mice transplanted with hepatocytes electroporated with Hpd-Cas9 RNP.

Supplementary Figure 7. Representative Masson's trichrome stained liver histology for *Fah*$^{-/-}$ mice transplanted with electroporated hepatocytes incubated in cytokine media.

Supplementary Figure 8. IHC staining against Hpd in Fah-/- mice transplanted with hepatocytes electroporated with Hpd-Cas9 RNP and mRNA.

Supplementary Figure 9. Gross liver images of Fah-/- recipient mice transplanted with hepatocytes electroporated with Hpd-Cas9 RNP and mRNA.

Supplementary Figure 10. Progressive weight data of *Fah*$^{-/-}$ mice transplanted with hepatocytes electroporated with Hpd-Cas9 RNP or mRNA.

Supplementary Figure 11. IHC staining images of liver section from *Fah*$^{-/-}$ mice transplanted with 500,000 viable hepatocytes after electroporation.

Supplementary Figure 12. Liver panel for *Fah*$^{-/-}$ mice transplanted with 500,000 viable hepatocytes electroporated with *Hpd*-Cas9 RNP.

Supplementary Figure 13. Representative H&E and Masson's trichrome stained liver histology images for *Fah*$^{-/-}$ mice transplanted with 500,000 viable hepatocytes electroporated with *Hpd*-Cas9 RNP.

Supplementary Figure 14: RNA expression of oxidative response and hepatocellular carcinoma genes in Fah-/- mice transplanted with 500,000 viable hepatocytes electroporated with Hpd-Cas9 RNP.

Supplementary Tables

Supplementary Table 1. PCR primers for amplification of *Hpd* for on-target TIDE analysis.

Supplementary Table 2. Histological assessment of H&E-stained histology images of the liver.

Supplementary Table 3. Viability and number of hepatocytes transplanted for each experiment.

Supplementary Methods

Hepatocyte electroporation

Hepatocytes were electroporated using 100 μL of Mouse/Rat Hepatocyte Nucleofector solution (Lonza) under the following conditions: 1×10^6 cells, 1.5 µL of 20 ng/µL *Hpd*-targeting sgRNA (Trilink Biotechnologies), and 4.9 µL of 61 µM SpCas9 V3 (Integrated DNA Technologies). The hepatocytes were immediately incubated on ice for 15 min, and 500 µL of ice-cold cytokine recovery medium was subsequently added to the cells and incubated for an additional 15 min on ice. After incubation, the required number of cells were centrifuged and resuspended in an ice-cold HMX medium for transplantation. When plated, electroporated hepatocytes were maintained in HMX medium in 6-well Collagen I-coated plates (Gibco) and cultured at 37°C in a humidified incubator with 5% CO_2 and ambient oxygen levels. After attachment, the medium was replaced with the Hepatocyte Maintenance Medium (Lonza). At 24 h after plating, a 0.25 mg/mL matrigel basement membrane matrix (Corning) was added as an overlay.

RNA expression analysis

Unique Dual-index Barcoded libraries for RNA-Seq were constructed using Tecan Universal Plus Total RNA-Seq with NuQuant Library kit (Tecan) with Tecan Mouse AnyDeplete (Tecan) rRNA depletion step following the manufacturer's recommendations. The libraries were quantified using a Qubit 1X dsDNA High Sensitivity assay (Invitrogen) on a Qubit 4 Fluorometer (Invitrogen) and qualified using a High Sensitivity D1000 DNA ScreenTape (Agilent) on an Agilent TapeStation 4150 (Agilent). The final libraries were diluted, and an equimolar pool was prepared according to the manufacturer's protocol. Pooled libraries were sequenced using an Illumina NovaSeq 6000 S1 v1.5 (300 cycles) flow cell (Illumina) with a 2×150 sequencing

chemistry to a depth of ~30 million reads per sample. The S1 flow cell data were demultiplexed using the Tecan NuQuant Library Dual Index barcodes and Illumina bcl2fastq2 software. Individual lane data from the S1 flow cell were merged for each sample and filtered for low-quality and short reads using the FASTP pre-processing workflow. [1] Residual rRNA contamination was filtered using the bbduk command from the BBMAP software package [2] and a custom rRNA multi-species reference generated from the SILVA database. [3] High-quality non-rRNA reads were aligned to the Mus musculus reference genome version 39 (GRCm39 GCF_000001635.27) using the GMAP_GSNAP aligner. [4] Expression quantification at the gene feature level was performed using the alignment files and the feature counts command from the subreads package. [5] A local cluster instance of the iDEP web application [6] was used for all downstream statistical analyses. Expression data were first normalized using regularized log transformation (rlog) with the following filtering criterion: minimum counts per million of two in at least two libraries. Rlog-transformed data were used for quality assessments, MDS plots, and bar plot visualizations. Raw read counts were assessed for differential expression using the DESeq2 pipeline [7] and a single factor negative binomial generalized linear model (Y ~ G + e) with three levels (Cas9 RNP, Wild-type Control and NTBC-Off Control). All possible pairwise contrasts were assessed for the genes of interest using the Wald test. Genes with |Log2FC| > 1 and Benjamini-Hochberg's False Discovery Rate adjusted p-value < 0.05 were considered statistically significant.

1. Chen S, Zhou Y, Chen Y, Gu J. fastp: an ultra-fast all-in-one FASTQ preprocessor. *Bioinformatics*. Sep 1 2018;34(17):i884-i890. doi:10.1093/bioinformatics/bty560
2. Bushnell B. *BBMap: a fast, accurate, splice-aware aligner*. 2014.
3. Quast C, Pruesse E, Yilmaz P, et al. The SILVA ribosomal RNA gene database project: improved data processing and web-based tools. *Nucleic Acids Res*. Jan 2013;41(Database issue):D590-6. doi:10.1093/nar/gks1219

4. Wu TD, Reeder J, Lawrence M, Becker G, Brauer MJ. GMAP and GSNAP for Genomic Sequence Alignment: Enhancements to Speed, Accuracy, and Functionality. *Methods Mol Biol*. 2016;1418:283-334. doi:10.1007/978-1-4939-3578-9_15
5. Liao Y, Smyth GK, Shi W. The Subread aligner: fast, accurate and scalable read mapping by seed-and-vote. *Nucleic Acids Res*. May 1 2013;41(10):e108. doi:10.1093/nar/gkt214
6. Ge SX, Son EW, Yao R. iDEP: an integrated web application for differential expression and pathway analysis of RNA-Seq data. *BMC Bioinformatics*. Dec 19 2018;19(1):534. doi:10.1186/s12859-018-2486-6
7. Love MI, Huber W, Anders S. Moderated estimation of fold change and dispersion for RNA-seq data with DESeq2. *Genome Biol*. 2014;15(12):550. doi:10.1186/s13059-014-0550-8

Supplementary Figures

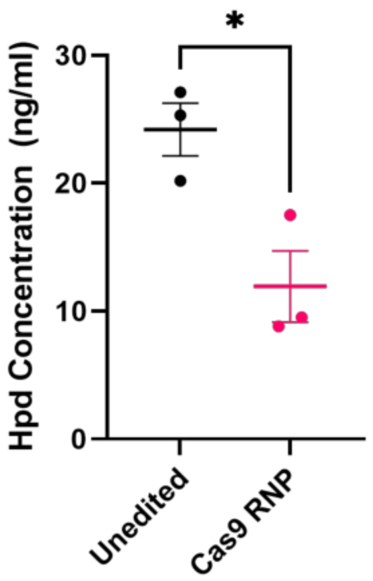

Supplementary Figure 1. Hpd concentration after electroporating *Hpd*-Cas9 into Hepa 1-6 cells. Hpd concentration measured using Hpd ELISA assay at 24 hours after electroporation. The mean levels are 24.2 and 11.9 for unedited and Cas9 RNP treated cells respectively. Dots represent different electroporation experiments and horizontal bars represent the means (n = 3). Statistical significance is indicated by asterisks, * is for $P < 0.05$.

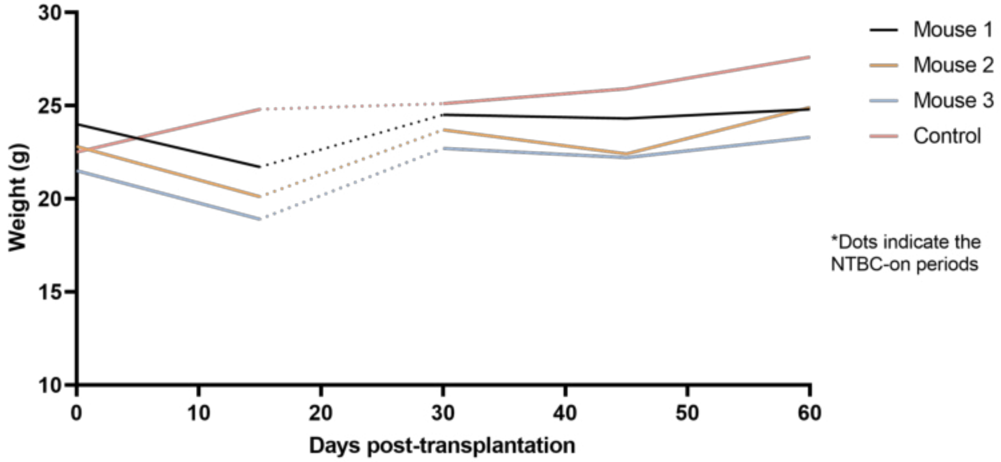

Supplementary Figure 2. Weight data of *Fah*$^{-/-}$ mice transplanted with unedited wild-type hepatocytes. Wild-type hepatocytes were isolated from GFP mice and transplanted into Fah$^{-/-}$ recipients. The recipient mice were taken off NTBC to stimulate in vivo selection of engrafted wild-type hepatocytes in the liver. Dotted lines represent periods on NTBC and solid lines represent NTBC-off periods.

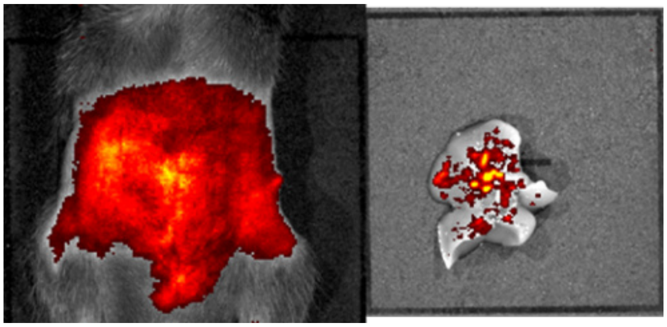

Supplementary Figure 3. Representative IVIS images of *Fah*$^{-/-}$ recipient mice transplanted with wild-type GFP hepatocytes. Whole body image was taken at 30 days post-transplantation and gross liver image was taken at 60 days post-transplantation. Camera IS1019N5225, Andor, iKon, Color scale min=4.95e6 max=2.40e7.

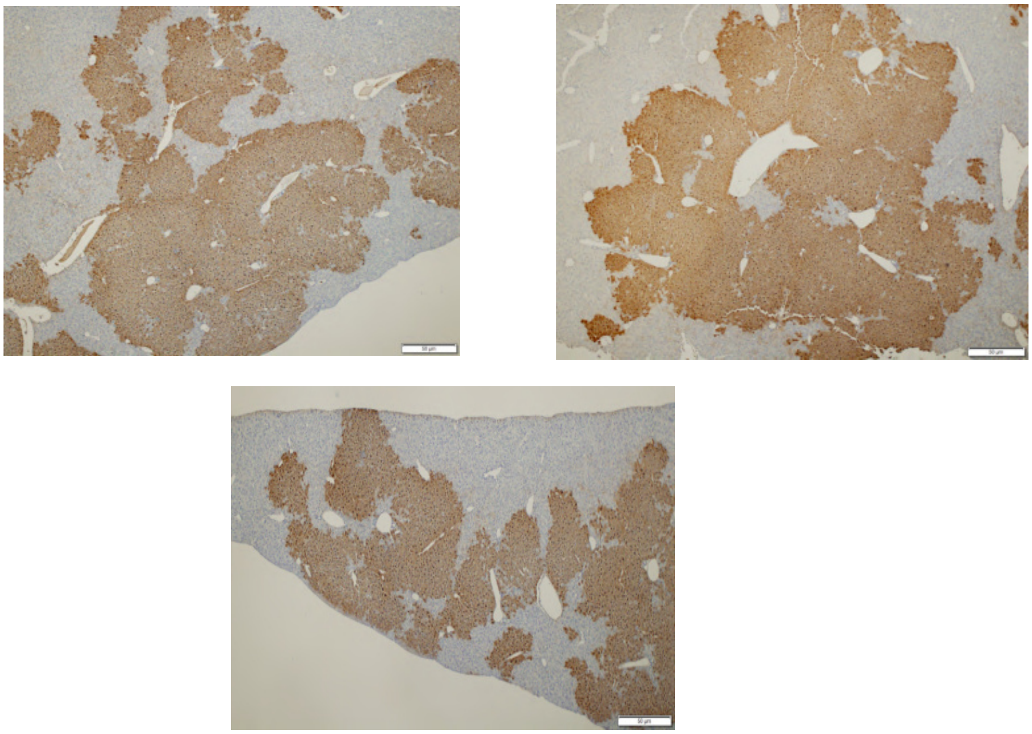

Supplementary Figure 4. IHC staining against Fah in *Fah*$^{-/-}$ mice transplanted with wild-type GFP hepatocytes. Recipient Fah$^{-/-}$ mice were transplanted with unedited wild-type hepatocytes. Staining was performed in liver tissue sections from mice sacrificed at 60 days post-transplantation. Scale bar represents 50 μm.

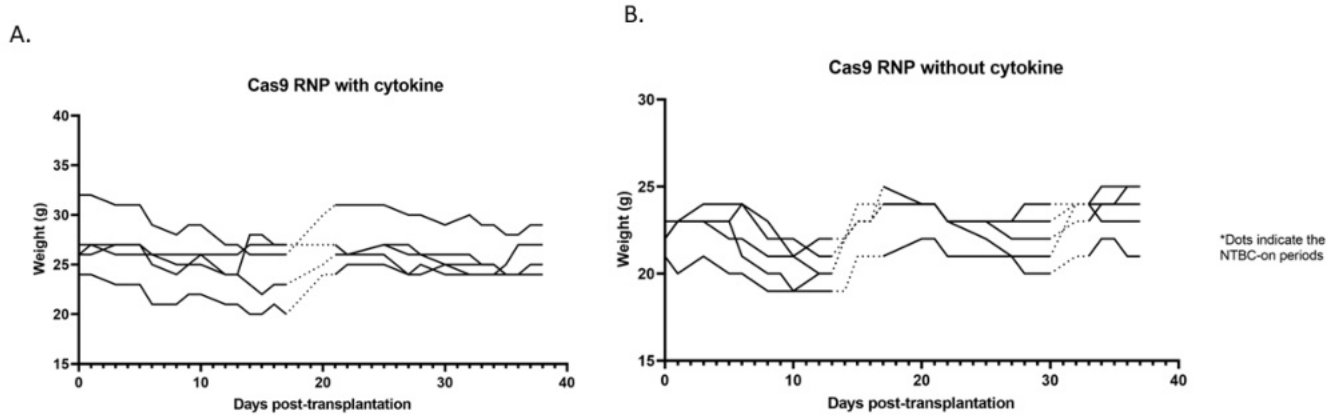

Supplementary Figure 5. Progressive weight data of *Fah*$^{-/-}$ mice transplanted with hepatocytes electroporated with *Hpd*-Cas9 RNP with or without the cytokine recovery media. (**A**) Weight data of mice transplanted with Cas9 RNP edited hepatocytes incubated in cytokine recovery media after electroporation. (**B**) Weight data of mice transplanted with Cas9 RNP edited hepatocytes incubated in plain HMX media without cytokines after electroporation. The dotted lines indicate the NTBC-on periods and the solid lines represent periods off NTBC.

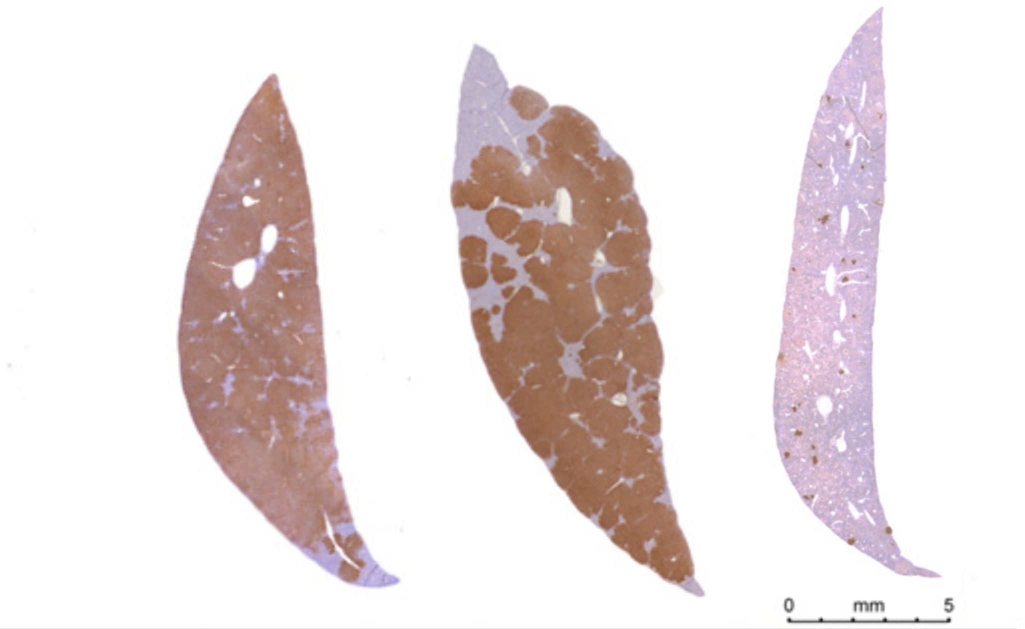

Electroporation	-	+	+
Cytokine incubation	-	+	-

Supplementary Figure 6. IHC staining against Fah in liver section from *Fah*$^{-/-}$ mice transplanted with hepatocytes electroporated with Hpd-Cas9 RNP. Donor hepatocytes were isolated from wild-type Fah-positive C57BL/6J mice. Legend below images indicate the treatment in hepatocytes prior to transplantation. Brown areas represent the wild-type Fah-positive hepatocytes engrafted in the liver tissue. The left image represents the liver tissue from mice transplanted with unedited control hepatocytes. The middle image represents the liver tissue from mice transplanted with electroporated hepatocytes incubated in cytokine recovery media. The image on the far right represents the liver tissue from mice transplanted with electroporated hepatocytes that were incubated in plain HMX media without cytokines.

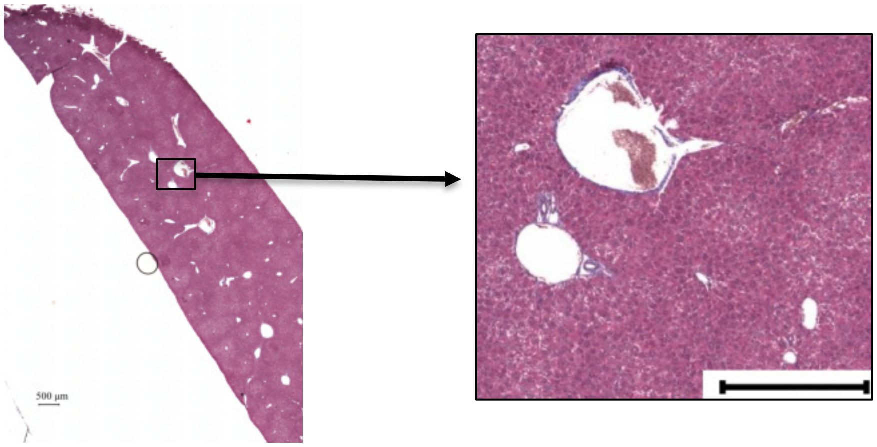

Supplementary Figure 7. Representative Masson's trichrome stained liver histology image for *Fah*$^{-/-}$ mice transplanted with electroporated hepatocytes incubated in cytokine recovery media. Scale bar represents 500 μm.

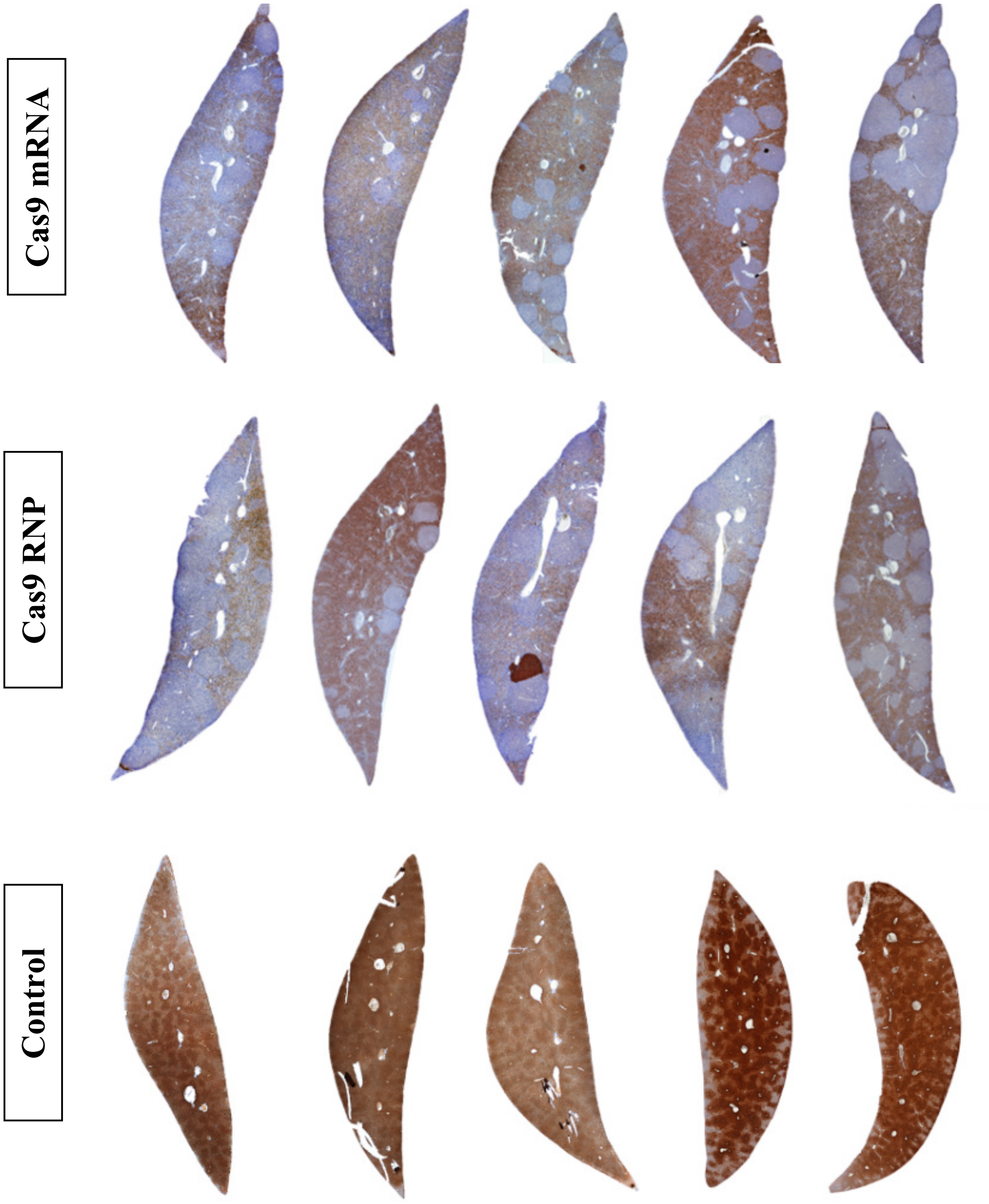

Supplementary Figure 8. IHC staining against Hpd in *Fah*$^{-/-}$ mice transplanted with hepatocytes electroporated with *Hpd*-Cas9 RNP and mRNA. Hepatocytes were isolated from *Fah*$^{-/-}$ mice, followed by electroporation with *Hpd*-Cas9 RNP or mRNA, and transplanted at a dose of 500,000 total cells into *Fah*$^{-/-}$ recipient mice. The top row shows stained liver tissue sections from recipients transplanted with hepatocytes edited with Cas9 mRNA while the middle row shows stained liver sections from recipients transplanted with Cas9 RNP. The bottom row shows stained liver sections from recipient mice transplanted with the unedited hepatocytes as controls.

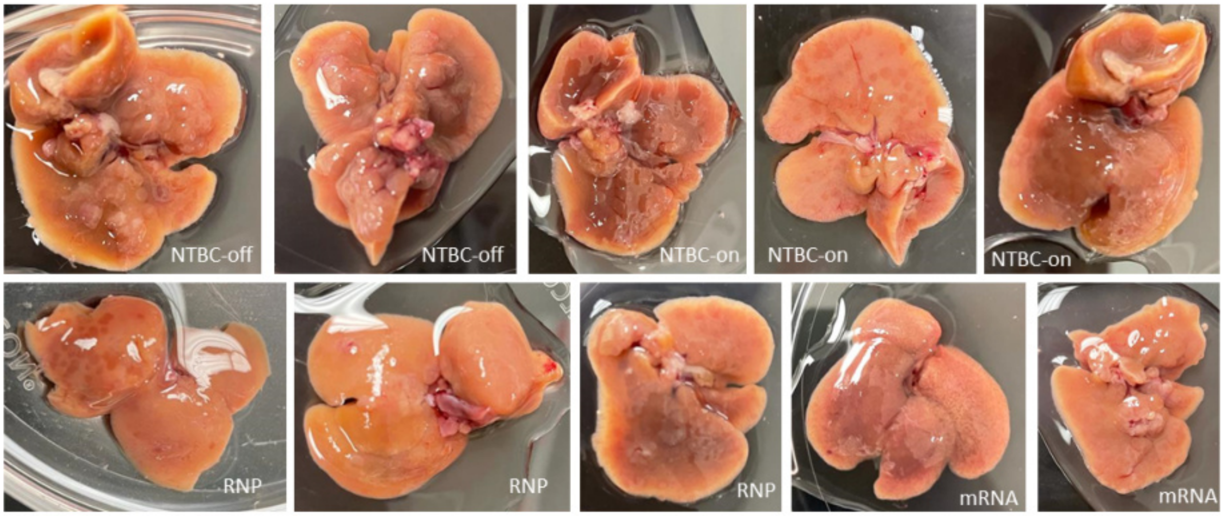

Supplementary Figure 9. Gross liver images of *Fah*$^{-/-}$ recipient mice transplanted with hepatocytes electroporated with Hpd-Cas9 RNP and mRNA. Hepatocytes were isolated from *Fah*$^{-/-}$ mice, followed by electroporation with *Hpd*-Cas9 RNP or mRNA, and transplanted into *Fah*$^{-/-}$ recipient mice. The top row shows the controls, including the *Fah*$^{-/-}$ mice that were kept on NTBC and off NTBC for the duration of the experiment. The bottom row shows liver images from experimental *Fah*$^{-/-}$ mice transplanted with hepatocytes electroporated with *Hpd*-Cas9 RNP or mRNA. The pictures were taken immediately after mice were sacrificed.

A.

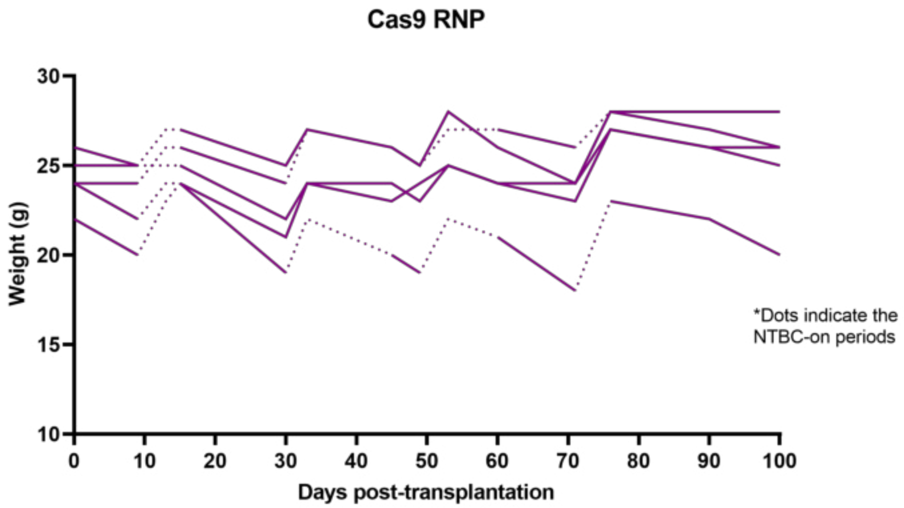

B.

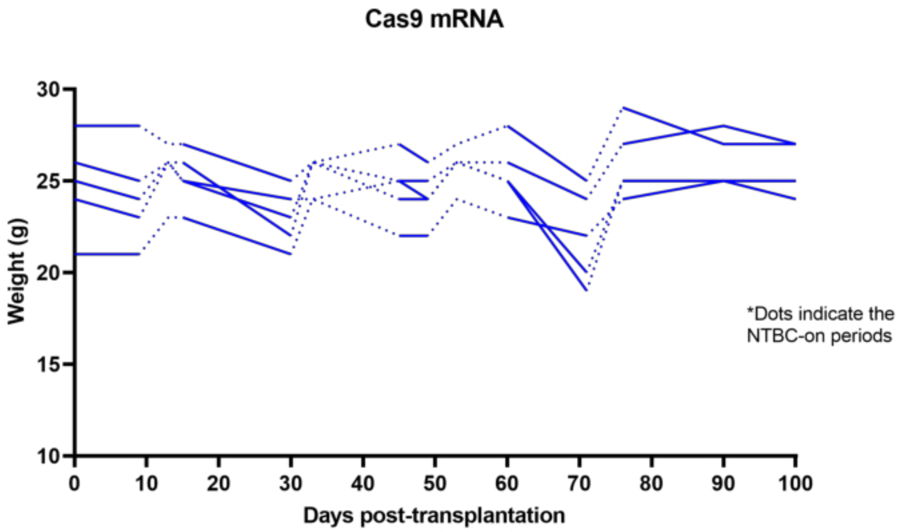

Supplementary Figure 10. Progressive weight data of *Fah*$^{-/-}$ mice transplanted with hepatocytes electroporated with *Hpd*-Cas9 RNP or mRNA. (**A**) Weight data of *Fah*$^{-/-}$ mice transplanted with diseased hepatocytes electroporated with *Hpd*-Cas9 RNP or (**B**) mRNA. Dotted lines indicate the NTBC-on periods, and the solid lines represent periods off NTBC.

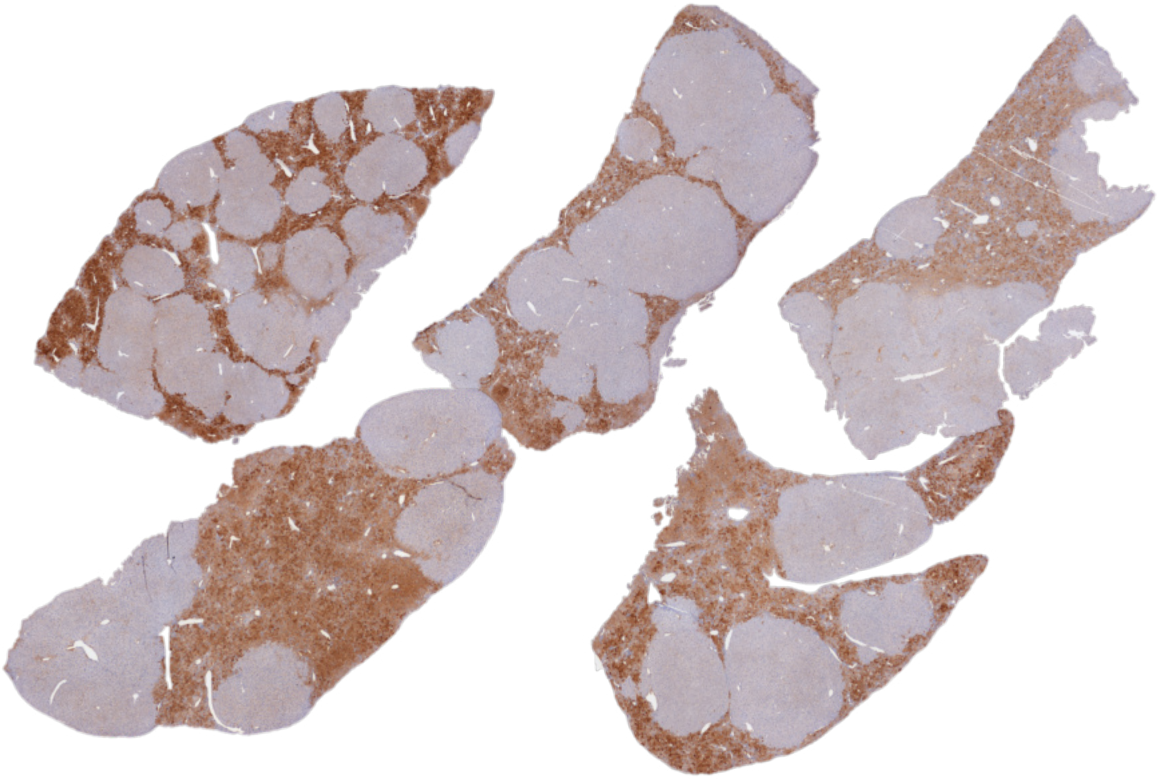

Supplementary Figure 11. IHC images of liver sections stained against Hpd from *Fah*$^{-/-}$ mice transplanted with 500,000 viable hepatocytes electroporated with *Hpd*-Cas9 RNP. Regions stained by anti-Hpd antibodies are indicated by the brown areas. The unstained pale areas represent the Hpd-negative hepatocytes edited by Cas9 RNP.

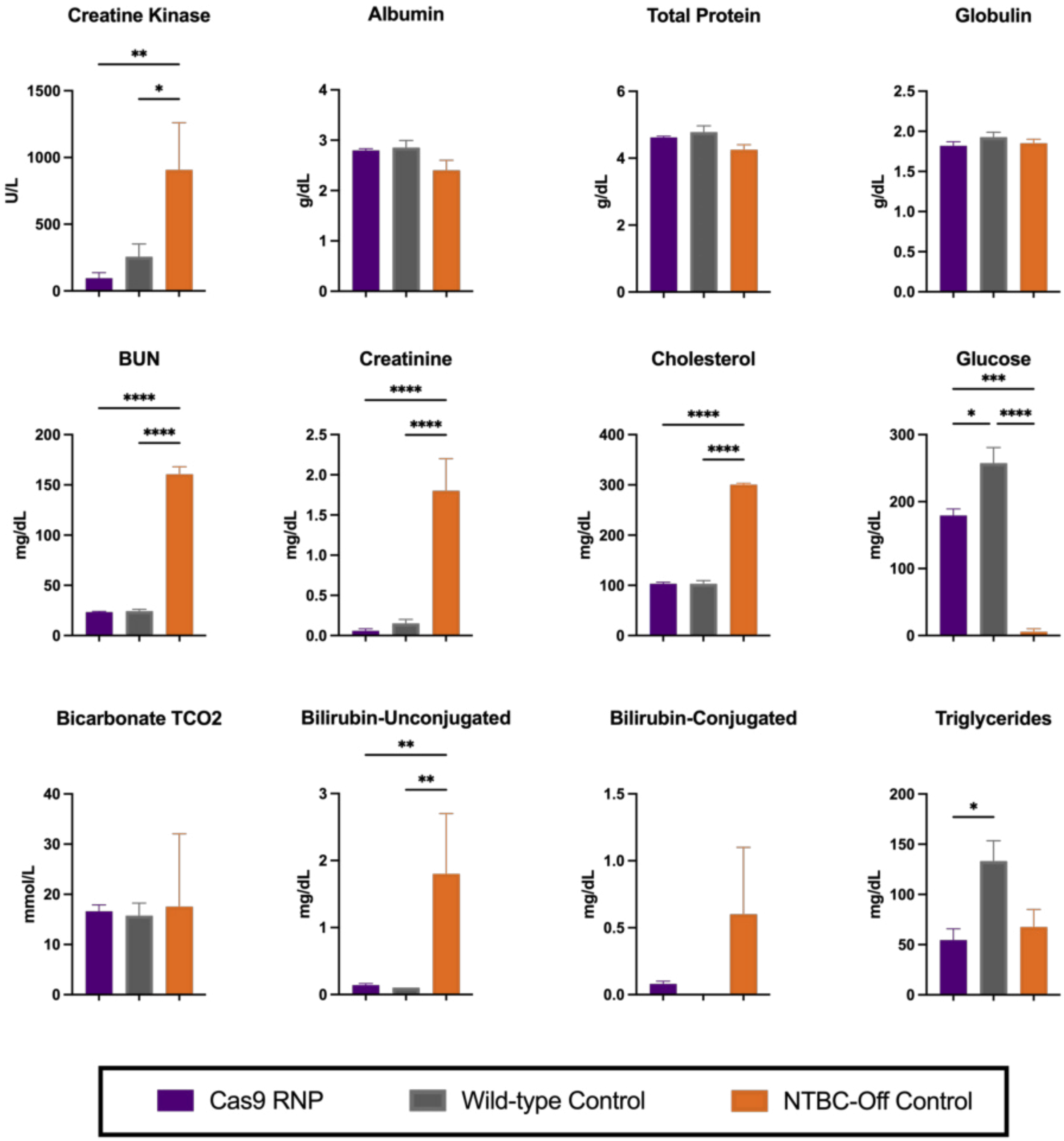

Supplementary Figure 12. Liver panel for *Fah*$^{-/-}$ mice transplanted with 500,000 viable hepatocytes electroporated with *Hpd*-Cas9 RNP. Results from liver function test performed on serum collected from *Fah*$^{-/-}$ recipients transplanted with 500,000 viable cells (n = 5). Controls consisted of untreated wild-type C57BL/6J mice (n = 4) and *Fah*$^{-/-}$ mice kept off NTBC (n = 2). Error bars represent the SEM. Differences are not significant unless indicated. Levels of significance *P < 0.05, **P < 0.01, ***P < 0.001, ****P < 0.0001 (one-way ANOVA with Tukey's multiple comparison).

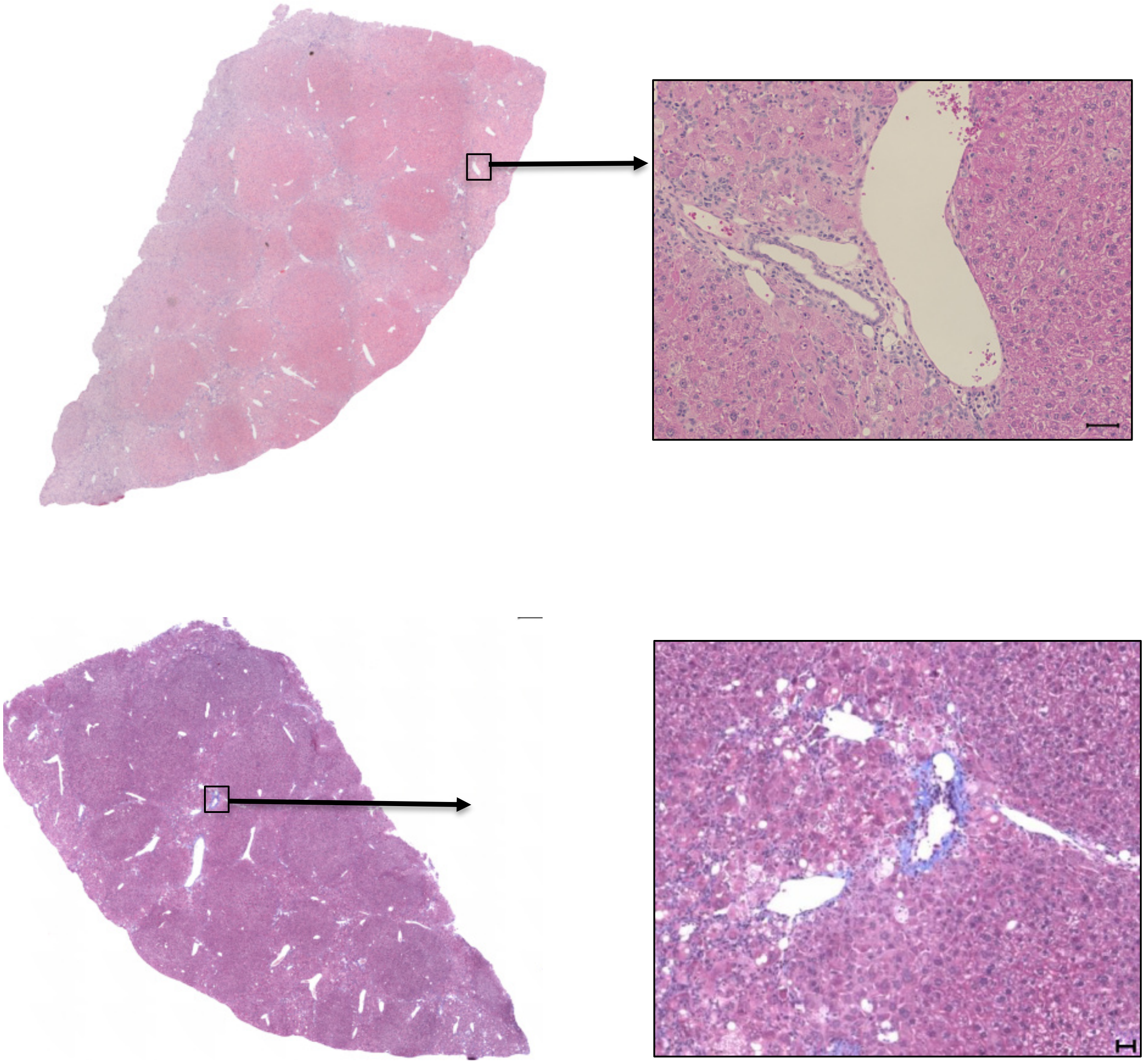

Supplementary Figure 13. Representative H&E and Masson's trichrome stained liver histology images for *Fah*$^{-/-}$ mice transplanted with 500,000 viable hepatocytes electroporated with *Hpd*-Cas9 RNP. The top row is the H&E-stained histology images, and the bottom row is the Masson-trichrome stained images. The scale bars represent 50 μm.

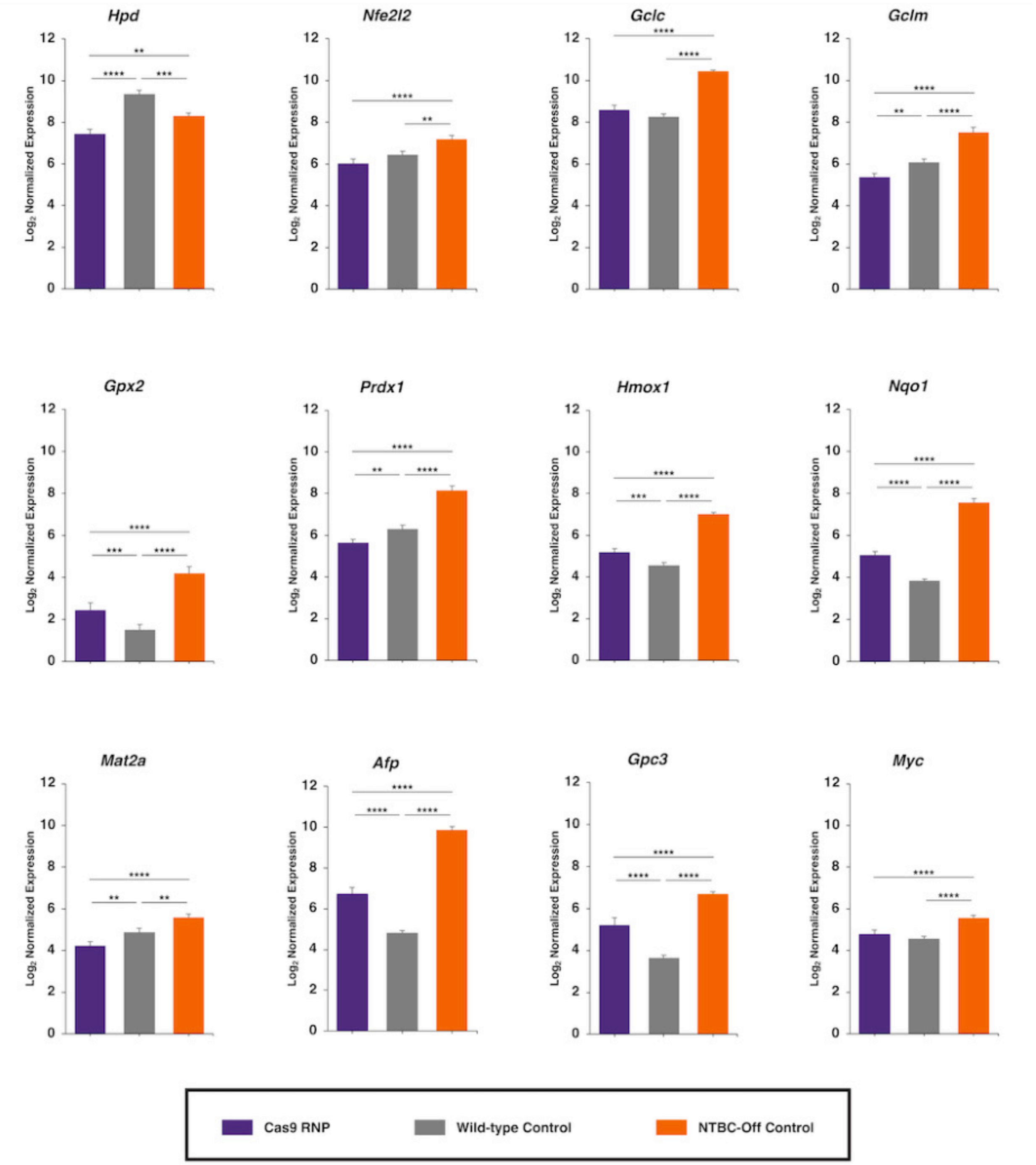

Supplementary Figure 14: RNA expression of oxidative response and hepatocellular carcinoma genes in *Fah*$^{-/-}$ mice transplanted with 500,000 viable hepatocytes electroporated with *Hpd*-Cas9 RNP. Normalized gene expression in *Fah*$^{-/-}$ mice transplanted with *Hpd*-Cas9 treated hepatocytes (N = 5). Controls consisted of untreated wild-type C57BL/6J mice (N = 4) and *Fah*$^{-/-}$ mice kept off NTBC (N = 3). Samples were analyzed in technical duplicates. Error bars represent the SEM. Differences are not significant unless indicated. Levels of significance are based on Benjamini-Hochberg's FDR-adjusted P-values: * P < 0.05, ** P < 0.01, *** P < 0.001, **** P < 0.0001 (Negative Binomial GLM with Wald test).

Supplementary Tables

Supplementary Table 1. PCR primers for amplification of *Hpd* for on-target TIDE analysis.

**FWD**	**5’-GGTCACCCATACTGTTCTCACG-3’**
**REV**	**5’-AGTCCTAGCCTGGCCTGGAT-3’**

Supplementary Table 2. Histological assessment of H&E-stained histology images of the liver.

Treatment	Steatosis	Fibrosis	Inflammation
**Transplanted with wild-type untransfected cells**	0	0	Minimal to mild portal & lobular
**Transplanted with wild-type electroporated cells incubated in cytokine media**	0	0	Mild portal & lobular
**Not transplanted with any cells and kept off NTBC**	2-25%, predominantly macrovesicular	0	Minimal portal & lobular
**Not transplanted with any cells and kept on NTBC**	0	0	Minimal portal & lobular
**Transplanted with *Fah* -/- cells electroporated with Cas9 RNP**	0-2%, predominantly macrovesicular	0	Minimal portal & lobular
**Transplanted with *Fah* -/- cells electroporated with Cas9 mRNA**	0-2%, predominantly macrovesicular	0	Mild portal & lobular

Supplementary Table 3. Viability and number of hepatocytes transplanted for each experiment.

Experiment	Viability of isolated cells	Viability after electroporation	Total number of cells transplanted	Number of viable cells transplanted
***Fah*$^{-/-}$ cells electroporated with Cas9 RNP vs mRNA**	77	70	500,000	350,000
***Fah*$^{-/-}$ cells electroporated with Cas9 RNP**	92	88	569,181	500,000
